# Genetic Variants in the Genes of the Stress Hormone Signalling Pathway and Depressive Symptoms during and after Pregnancy

**DOI:** 10.1155/2014/469278

**Published:** 2014-03-12

**Authors:** Michael Schneider, Anne Engel, Peter A. Fasching, Lothar Häberle, Elisabeth B. Binder, Franziska Voigt, Jennifer Grimm, Florian Faschingbauer, Anna Eichler, Ulf Dammer, Dirk Rebhan, Manuela Amann, Eva Raabe, Tamme W. Goecke, Carina Quast, Matthias W. Beckmann, Johannes Kornhuber, Anna Seifert, Stefanie Burghaus

**Affiliations:** ^1^Department of Gynecology and Obstetrics, University Hospital Erlangen, Friedrich-Alexander University Erlangen-Nuremberg, Erlangen, Germany; ^2^Max Planck Institute of Psychiatry, Munich, Germany; ^3^Department of Gynecology and Obstetrics, University Hospital Aachen, RWTH Aachen, Germany; ^4^Department of Child and Adolescent Mental Health, University Hospital Erlangen, Friedrich-Alexander University Erlangen-Nuremberg, Erlangen, Germany; ^5^Department of Psychiatry, University Hospital Erlangen, Friedrich-Alexander University Erlangen-Nuremberg, Erlangen, Germany

## Abstract

*Purpose*. The aim of this study was to investigate whether single nucleotide polymorphisms (SNPs) in genes of the stress hormone signaling pathway, specifically *FKBP5*, *NR3C1*, and *CRHR1*, are associated with depressive symptoms during and after pregnancy. *Methods*. The Franconian Maternal Health Evaluation Study (FRAMES) recruited healthy pregnant women prospectively for the assessment of maternal and fetal health including the assessment of depressiveness. The German version of the 10-item Edinburgh Postnatal Depression Scale (EPDS) was completed at three time points in this prospective cohort study. Visit 1 was at study entry in the third trimester of the pregnancy, visit 2 was shortly after birth, and visit 3 was 6–8 months after birth. Germline DNA was collected from 361 pregnant women. Nine SNPs in the above mentioned genes were genotyped. After construction of haplotypes for each gene, a multifactorial linear mixed model was performed to analyse the depression values over time. *Results*. EPDS values were within expected ranges and comparable to previously published studies. Neither did the depression scores differ for comparisons among haplotypes at fixed time points nor did the change over time differ among haplotypes for the examined genes. No haplotype showed significant associations with depressive symptoms severity during pregnancy or the postpartum period. *Conclusion*. The analysed candidate haplotypes in *FKBP5*, *NR3C1*, and *CRHR1* did not show an association with depression scores as assessed by EPDS in this cohort of healthy unselected pregnant women.

## 1. Introduction

During pregnancy the overall prevalence of depressive episodes is about 10–20% [1, 2] and about 6–15% in childbed [3, 4]. A total of about 70,000–90,000 women in Germany suffer from this disorder every year, with 5-6% developing major depression [2, 4, 5]. The prevalence compares to that of non-pregnancy-associated depression; however, new depression occurs more often during pregnancy and in the postpartum period [6].

There is a correlation of pregnancy-associated depression with poorer obstetric outcome measures, with fetal and neonatal complications [7, 8], with the length of the mother's hospital stay at the time of delivery [9], and with a negative impact on the child's development [10–13]. Information about the pathogenesis for pregnancy-associated depression may therefore be helpful for planning early interventions and understanding the pathogenesis of this disease, as it is not a part of the early intervention program in Germany yet [14]. In the general population, it is thought that between 33% and 77% of major depression can be attributed to genetic susceptibility [15, 16]. Several genome-wide association studies have been conducted [17–24] with some evidence for genetic susceptibility variants. Some studies described an association between perinatal depression and a family history of depression or perinatal depression [25–27]; however, only few studies have investigated specific genetic risk factors for perinatal depression.

One signalling pathway that is of specific interest in this context is the stress hormone system [28]. This signalling system is thought to be the key regulator of the response to environmental stressors. Its dysregulation is found consistently in stress related psychiatric disorders like major depression or posttraumatic stress disorder [29–31] and might play a relevant role in pregnant women [32]. With regard to pregnancy-associated depression, stress appeared to be one of the most stable factors in multivariate models for the prediction of depression during pregnancy [33], making this topic interesting for further research concerning this phenotype. Furthermore in utero exposure to stress and its subsequent exposure to glucocorticoids are discussed to have an influence on the development of behavioural stress response in the offspring [34, 35].

This study focuses on three genes, for which it is has been shown that genetic variants are associated with depressive symptomatology, especially in the context of stressful or adverse life events: the genes encoding the corticotropin releasing factor receptor 1 (*CRHR1*), the glucocorticoid receptor (*NR3C1*), and FK506 binding protein 51 (*FKBP5*).

CRHR1 function has been reported to be specifically associated with increased fear, alertness, depression, and anxiety [36–39]. Genetic variants in* CRHR1* have been associated with anxiety disorders, major depression, and alcoholism, especially in the context of early life adverse events [28, 40].

*NR3C1* encodes the glucocorticoid receptor (GR). GR signalling has been reported to be disrupted in both depression and anxiety disorders [30, 41]. Several genetic variants have been described to result in functional changes of the GR [42, 43].

FKBP5 is known to bind to and alter the function of steroid hormone receptors, including the GR [44] and is a negative feedback regulator of GR function [31, 45]. Functional genetic variants in FKBP5 have been described to alter stress hormone response regulation as well as the risk to suffer from depression and other psychiatry disorders when exposed to childhood trauma [28, 46].

The aim of the present study was to test whether genetic variants in* FKBP5*,* GR (NR3C1)*, and* CRHR1*, previously described to increase the risk for depression, are associated with longitudinal measures of depressive symptoms in a cohort of pregnant women assessed in the third trimester of pregnancy, 2-3 days and 6 months after delivery.

## 2. Patients and Methods

### 2.1. Patient Selection and Biomaterial Retrieval

The Franconian Maternal Health Evaluation Study (FRAMES) is a prospective study, which recruited pregnant women from 2005 to 2007. Aim was the investigation of risk factors for pregnancy-associated depression [4, 47–49]. Previously we presented the influence of variants in* TPH2* on depression measurement scores during the pregnancy [47] and that of variants in the serotonin transporter* 5-HTTLPR* on different depression levels after childbirth with regard to lifetime and current psychological stressors [50].

Inclusion criteria were age of 18 years or older with an intact pregnancy and at a gestational age of at least 31 weeks. They were invited to participate when they presented to register for the upcoming birth. A total of 1100 women were prospectively included. Assessment of genetic risk factors for postpartum depression was included as a study aim after the recruitment of women was completed in 2008. Blood samples for genetic analysis were therefore not taken prospectively, and the women had to be recalled for this purpose. This took place between January 2008 and July 2008. The patients were contacted by phone and invited to undergo blood sampling and take part in the genetic association study. From the primary study population (*n* = 1100) current phone numbers could be determined from 780 patients and 705 could be reached. 130 women declined to take part; the rest was appointed for a blood draw. Women, who did not show up, were contacted again and offered another appointment. A total of 431 women presented for blood sampling (final study population). DNA extraction was successful in 423 cases. DNA was considered unsuitable for the study if the DNA concentration was below 30 ng/*μ*L according to the PicoGreen DNA concentration measurements. In addition, 62 women had to be excluded from the analysis because the depression measurement was lacking for at least one time point in the study, resulting in a final sample size for this study of 361 patients. The study was approved by the Ethics Committee of the Medical Faculty of Friedrich-Alexander University of Erlangen-Nuremberg and all of the patients provided written informed consent.

### 2.2. Questionnaire

The participants were interviewed using standardised 10-item Edinburgh Postnatal Depression Scale (EPDS) questionnaires, in the German version [51], at three time points: prepartal, from the 31st week of pregnancy onwards (Q1); 48–72 hours postpartum (Q2) to capture the initial phase of the maternity blues; and 6–8 months after birth (Q3). Additionally a structured questionnaire was used to document common epidemiological parameters and medical history that was not documented in the patients' files. This questionnaire included the question about preexisting psychiatric disorders, which was an exclusion criteria for this study. The first two questionnaires (Q1, Q2) were structured as personal interviews using standardised manuals, which were conducted by trained and medically qualified staff. The third questionnaire (Q3) was carried out by phone interview. The reliability of phone questionnaires in this setting can be regarded as confirmed [52].

### 2.3. SNP Selection

SNPs in the genes* FKBP5*,* NR3C1*, and* CRHR1* have been selected for genotyping based on published positive association studies with depression or depressive symptoms for the respective SNPs and haplotypes (see Section 1). The SNPs with the strongest gene environment interaction effects, which mean depressive symptoms, were selected (*CRHR1* SNPs: rs7209436 and rs110402 [28, 40];* NR3C1* SNPs: rs41423247, rs6195, and rs10482605 [42, 43];* FKBP5* SNPs: rs1360780, rs9296158, rs3800373, and rs9470080 [28, 46]). SNP IDs and their minor allele frequency (MAF) are reported in Table 1.

### 2.4. DNA Preparation and Genotyping

DNA was extracted from 10 mL of ethylenediaminetetraacetic acid (EDTA) blood using the Puregene whole-blood DNA extraction kit (Gentra Systems, Minneapolis, MN, USA).* FKBP5*,* NR3C1*, and* CRHR1* SNPs were analysed on a Sequenom platform using the iPlex technology (Sequenom, San Diego, CA, USA) in a multiplex assay using 10 ng of DNA. For quality control, duplicate DNAs as well as negative controls were included in the genotyping plates. Genotype calls were made using the ArrayTyper 3.4 software (Sequenom, San Diego, CA, USA).

### 2.5. Statistical Considerations

Genotypes were analysed as haplotypes. The reconstruction of haplotypes was carried out with an expectation-maximisation (EM) algorithm [53]. For the haplotype reconstruction, all SNPs were grouped by gene. Genotype distributions were tested for Hardy-Weinberg Equilibrium. Haplotypes were examined rather than single SNPs because haplotypes may provide more genetic information. Associations between SNPs and outcome measure are expected to be reflected in associations between haplotypes and outcome measure, but not necessarily vice versa.

The EPDS value was regarded as continuous measurement with a range from 0 to 26. Depression values from the three different time points Q1, Q2, and Q3 were compared. For each haplotype, a categorical variable with levels according to the frequency of 0, 1, or 2 copies per patient was generated. Small groups with fewer than five carriers of two copies of a haplotype were joined with the carriers of one copy. Extremely rare haplotypes with an overall haplotype frequency of fewer than 10 occurrences were excluded from the analysis. Consideration of the haplotypes as ordinal variables was rejected due to nonlinear coherence with EPDS.

The association between haplotypes and the course of depression was analysed using linear mixed models with EPDS as target variable. For each haplotype block, a linear mixed model was fitted with patient as random effect and haplotypes, time (Q1, Q2, Q3), and the interactions of haplotypes by time as fixed effects. These linear models were each compared with a basic linear mixed model with patient as random effect and time as the only fixed effect, using the likelihood ratio test. A significant test result means that the haplotypes are associated with EPDS. In that case the linear model was further analysed using *F*-tests of fixed effects. The *P* values of the likelihood ratio tests were adjusted for multiple testing according to the method of Bonferroni-Holm.

The model requirements (e.g., normal distribution of the standardised residuals) were tested graphically. No replacement of missing data took place. The random effect “patient” takes into account the fact that each patient had repeated EPDS measures. The models were fitted by maximum likelihood (ML) instead of restricted maximum likelihood (REML) in order to apply likelihood ratio tests to models with different fixed effects. A sensitivity analyses showed that both estimation methods gave almost identical results.

All of the tests were two-sided, and a *P* value of <0.05 was regarded as statistically significant. The statistical analyses were carried out using the R system for statistical computing (version 2.13.1; R Development Core Team, Vienna, Austria, 2011) and the SAS software package (version 9.2, SAS Institute, Inc., Cary, NC, USA).

## 3. Results

**Figure 1:**
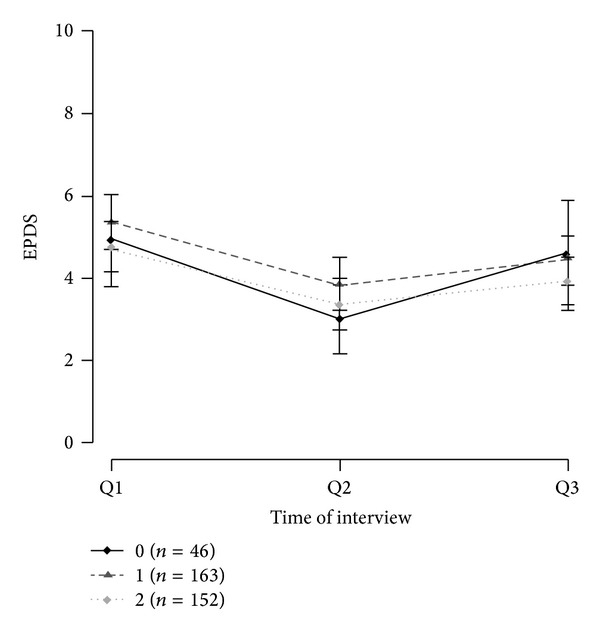
Mean EPDS values of FKBP5 haplotype CGTC with 95% confidence intervals.

**Figure 2:**
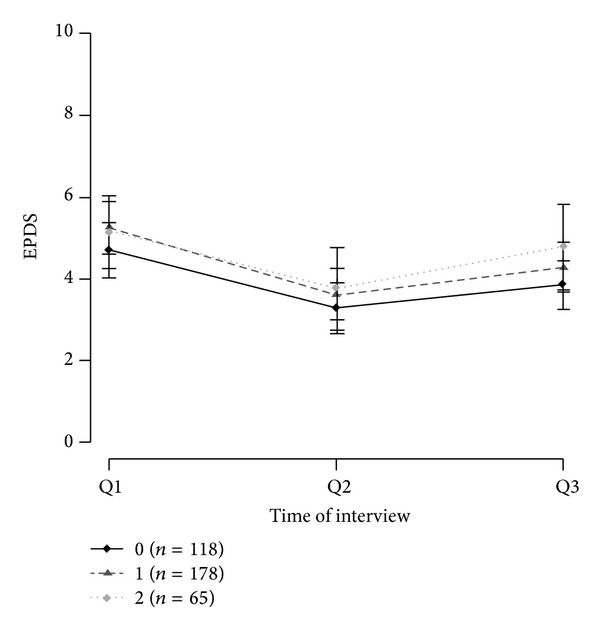
Mean EPDS values of GRNR3C1 haplotype GAT with 95% confidence intervals.

**Figure 3:**
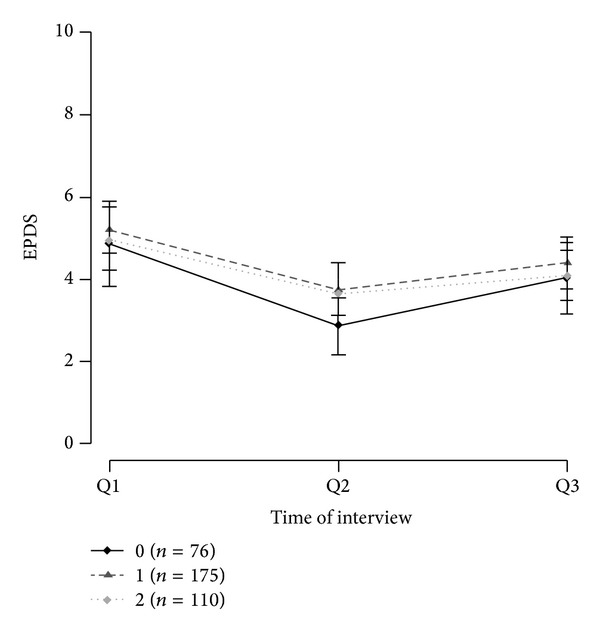
Mean EPDS values of CRHR1 haplotype CC with 95% confidence intervals.

The genotype frequency and allele distributions are shown in Table 1. The genotype distribution for all SNPs was consistent with the Hardy-Weinberg equilibrium (*P* = 0.16 for rs10482605; *P* between 0.56 and 1.00 for the other SNPs). The distributions and frequencies for each haplotype block are presented in Table 2. For the most frequent haplotype of each gene the mean EPDS values of Q1, Q2, and Q3 are shown in Figures 1, 2, and 3 for carriers of 0, 1, or 2 copies of the respective haplotypes.

The SNPs within gene* FKBP5* formed ten haplotypes, but only four of them, CGTC, CGTT, TATT, and TAGT, occurred with a frequency usable for analysis. For haplotype CGTT the group of carriers of two copies (0.28%) was joined with the carriers of one copy (5.26%).

Haplotype reconstruction with the* NR3C1* SNPs resulted in five haplotypes. The most common haplotype was GAT, with 49.31% of patients carrying two copies and 32.69% carrying one copy. For haplotype GGT the group of carriers of two copies (0.28%) was joined with the carriers of one copy (9.14%). The haplotype CAC had to be discarded because of only two occurrences.

The haplotype reconstruction within gene* CRHR1* resulted in three haplotypes where haplotype CC was the most common with 30.47% carrying two copies and 48.48% carrying one copy.

None of the haplotypes showed a significant result for the likelihood ratio test (unadjusted *P* values: FKBP5, *P* = 0.45; NR3C1, *P* = 0.78;* CRHR1*, *P* = 0.61). Therefore no further analysis was performed, as differences between genotype groups at one time point or over different time points cannot be assumed.

## 4. Discussion

With our association study in a cohort of pregnant women without further risk factors for depressive or anxiety disorders, we could not show that candidate single nucleotide polymorphisms within the genes* FKBP5*,* NR3C1*, and* CRHR1* are associated with EPDS values during or after pregnancy.

The candidate genes were selected because of their role within stress hormone signalling system which is one of the possible mediators between environmental stressors and the development of a depressive reaction. Several genetic factors have been discovered that explain individual responses to stressful events [28, 29, 54–56].

However, with our study design and the examined genetic variants, no effect on EPDS values could be seen, neither between haplotypes at specific time points, nor in comparing the changes of EPDS over time according to haplotypes. Several factors specific to this study will be discussed below.

As we were studying a cohort of women with an uncomplicated pregnancy and no prior history of psychiatric disease, there might be a different genotype distribution in our cohort than in cohorts of women without the inclusion criterion of pregnancy. It was reported that variants in NR3C1 may have an influence on gonadotropin levels in women with anovulatory polycystic ovary syndrome (PCOS) [57]. Another study reported variants in NR3C1 to be associated with recurrent miscarriages [58]. There is further preclinical evidence that exposure to glucocorticoids leads to the apoptosis of fetal ovary germline cells, having possible impact on fertility [59]. Preselecting of women with an uncomplicated pregnancy could therefore result in a population with slightly different genotype distribution.

Furthermore during pregnancy many signalling pathways adapt with regard to ensuring the function of the pregnancy, with one of them being the hypothalamic-pituitary-adrenal axis or stress hormone system [60]. Therefore genetic associations that are observed in women without a pregnancy might not be found in a population of pregnant women. In particular during the third trimester progressively increasing circulating levels of placental CRH are seen [61] as well as gradually decreasing levels of CRH binding protein [62]. Maternal distress during pregnancy increases plasma levels of cortisol and CRH in addition to the already physiologically increased levels [63]. After delivery a central suppression of hypothalamic CRH secretion might explain a generally increased vulnerability to the affective disorders observed during this period [64]. For these reasons associations between genotypes and phenotypes might be different in pregnant and nonpregnant populations.

Several limitations of this study have to be taken into consideration. One might be the use of the EPDS questionnaire a few days after childbirth. The EPDS reflects the experience and mood state of women during the week before completing the questionnaire, intentionally skipping somatic symptoms that are associated with depression but appear quite often after delivery in healthy women, such as sleep disturbances or fatigue, and it has been validated for administration during pregnancy and a few weeks into the postpartum period [65–69]. The rating within the first days after delivery might thus also reflect the mood during the last days of pregnancy. Another limitation might be that patients were recontacted for blood sampling for DNA extraction after the end of the study. However, there were no differences with regard to prepartum or postpartum EPDS scores in women participating or not participating in the genetic substudy (data not shown). Women were screened for preexisting psychiatric disorders only by a questionnaire. This self-reported depression is not as accurate as the assessment by a formal psychiatric diagnostic interview. However, in contrast to other studies, a classic case/control design was not used for the analysis, and the prevalence of clinical depression was rather low in this cohort (6% as measured with the EPDS). Continuous EPDS values were therefore selected as the outcome variable in order to maximize the power of the study. Finally, due to the limited sample size our study might not show smaller effect of the examined genetic variants.

In conclusion we could not show an association between depression measurements as assessed by EPDS values during or after pregnancy and candidate haplotypes in the genes* FKBP5*,* NR3C1*, and* CRHR1*. As other studies have shown some association between genetic variants in these genes and depressive symptomatology, our null results could be explained by a small sample size or a generally different role of genetic variants in genes of the stress hormone signalling pathway in pregnant women.

## Figures and Tables

**Table 1:** Genotype and allele distribution for each single nucleotide polymorphism (SNP). Absolute frequencies and percentages (in brackets) are shown.

SNP	Chrom.^1^	Position	Alleles^2^	MAF^3^ (%)	Homozygous, common^4^	Heterozygous^4^	Homozygous rare^4^
rs1360780 (FKBP5)	6	35607571	C/T	32.0	168 (46.5)	155 (43.2)	38 (10.5)
rs9296158 (FKBP5)	6	35567082	G/A	32.0	166 (46.1)	156 (43.3)	37 (10.2)
rs3800373 (FKBP5)	6	35542476	T/G	28.4	185 (51.8)	147 (40.7)	29 (8.0)
rs9470080 (FKBP5)	6	35646435	C/T	34.7	155 (42.9)	160 (44.6)	45 (12.5)
rs41423247 (NR3C1)	5	142778575	G/C	34.7	156 (43.3)	158 (43.9)	46 (12.7)
rs6195 (NR3C1)	5	142779317	A/G	4.7	327 (91.6)	34 (9.4)	0 (0.0)
rs10482605 (NR3C1)	5	142783521	T/C	18.3	242 (67.0)	99 (27.6)	16 (4.4)
rs110402 (CRHR1)	17	43880047	C/T	45.3	110 (30.6)	175 (48.6)	76 (21.1)
rs7209436 (CRHR1)	17	43870142	C/T	44.0	115 (32.2)	174 (48.2)	72 (19.9)

^1^Chromosome; ^2^major/minor allele, based on the forward strand and minor allele frequency; ^3^minor allele frequency; ^4^frequency, percentage in brackets.

**Table 2:** Reconstructed haplotypes for each gene and absolute frequencies and percentages (in brackets).

No	Gene	SNP	Haplotype	Haplotype frequency		
				0	1	2
1	FKBP5	1–4	CGTC	46 (12.74%)	163 (45.15%)	152 (42.11%)
2			CGTT	341 (94.46%)	19 (5.26%)	1 (0.28%)
3			CATC	360 (99.72%)	1 (0.28%)	0 (0.00%)
4			CATT	360 (99.72%)	1 (0.28%)	0 (0.00%)
5			CAGC	360 (99.72%)	1 (0.28%)	0 (0.00%)
6			TGTT	358 (99.17%)	3 (0.38%)	0 (0.00%)
7			TATC	359 (99.45%)	2 (0.55%)	0 (0.00%)
8			TATT	339 (93.91%)	22 (6.09%)	0 (0.00%)
9			TAGC	360 (99.72%)	1 (0.28%)	0 (0.00%)
10			TAGT	187 (51.80%)	145 (40.17%)	29 (8.03%)

11	GR-NR3C1	5–7	GAT	118 (32.69%)	178 (49.31%)	65 (18.01%)
12			GAC	247 (68.42%)	99 (27.42%)	15 (4.16%)
13			GGT	327 (90.58%)	33 (9.14%)	1 (0.28%)
14			CAT	158 (43.77%)	158 (43.77%)	45 (12.47%)
15			CAC	359 (99.45%)	2 (0.55%)	0 (0.00%)

16	CRHR1	8+9	CC	76 (21.05%)	175 (48.48%)	110 (30.47%)
17			TC	352 (97.51%)	9 (2.49%)	0 (0.00%)
18			TT	115 (31.86%)	174 (48.20%)	72 (19.94%)
